# Dietary and Lifestyle Factors Serve as Predictors of Successful Weight Loss Maintenance Postbariatric Surgery

**DOI:** 10.1155/2019/7295978

**Published:** 2019-02-12

**Authors:** Afshan Masood, Lujain Alsheddi, Loura Alfayadh, Bushra Bukhari, Ruba Elawad, Assim A. Alfadda

**Affiliations:** ^1^Obesity Research Center, College of Medicine, King Saud University, P.O. Box 2925 (98), Riyadh 11461, Saudi Arabia; ^2^Clinical Nutrition Program, Department of Community Health Sciences, College of Applied Medical Sciences, King Saud University, P.O. Box 10219, Riyadh 11433, Saudi Arabia; ^3^Department of Medicine, College of Medicine, King Saud University, P.O. Box 2925 (38), Riyadh 11461, Saudi Arabia

## Abstract

Bariatric surgery is considered to be an effective treatment for the resolution of severe obesity; however, in more than half of the bariatric surgery patients, weight reacquisition occurs as early as 18 months postsurgery, compromising the surgery's beneficial effects. Maintaining weight loss after surgery poses a great challenge, necessitating the identification of predicting factors. In the present study, we explored the association between weight regain and dietary habits and behavioral lifestyle practices in patients following bariatric surgery. Fifty patients who underwent bariatric surgery with ≥18-month postoperative period of follow-up were included. They were classified into two groups: weight maintainers (*n* = 29) were patients who regained <15% of their weight, and weight regainers (*n* = 21) were patients who regained ≥15% of their weight compared to their lowest postoperative weight. The mean age of the study participants was 41.4 ± 8.9 years, and twenty-eight patients (56%) of the total, were females. A detailed analysis of dietary and lifestyle habits was performed by questionnaire-based interviews. Significant weight regain was noted in the regainers compared to the maintainers (19.6 ± 8.4 kg vs. 4.5 ± 3.5 kg, respectively, *P* ≤ 0.001), which was attributed to their following of unhealthy dietary habits and behavioral lifestyle practices. The dietary and behavioral lifestyle practices adopted by the maintainers were higher fiber consumption and water intake, monitored pace of eating, evasion of emotional binge, and distracted eating and following of self-assessment behaviors. Additionally, regular nutritional follow-ups and compliance with postoperative dietary counseling significantly helped to improve weight maintenance. In conclusion, the effectiveness of weight loss postbariatric surgery was compromised by weight regain due to unhealthy dietary and behavioral lifestyle practices stemming from a lack of nutritional guidance and knowledge. The implementation of comprehensive nutritional counseling and advice on behavioral changes before and after surgery will help achieve optimal weight results.

## 1. Introduction

Obesity and its associated comorbidities, including metabolic syndrome and type 2 diabetes, continue to represent a major cause of public health burden worldwide, and they are projected to reach approximately 58% of the population worldwide in the upcoming years [1]. The same is true for the Saudi population, where the prevalence rate of obesity has dramatically increased from 22% in 1993 to 36% in 2005 and is projected to rise to 71%, with one of three adults being obese and at least one of ten adults having morbid obesity [2, 3]. The treatment and remission of obesity through lifestyle modifications and pharmacotherapy has largely yielded limited results compared with surgical interventions, especially for morbidly obese patients [4, 5].

Bariatric surgery is currently considered one of the most effective treatment approaches for morbid and severe obesity. Compared with traditional nonsurgical weight loss methods, it achieves rapid, significant, and sustained weight loss along with the remission of obesity-related comorbidities, and despite its invasiveness, it is the most efficacious approach for the treatment of obesity and obesity-related comorbidities [6]. The procedures have evolved from invasive, open methods to minimally invasive laparoscopic procedures with mortality rates comparable to those of common general surgical procedures. In spite of their proven benefits, their long-term effects on sustaining weight loss have been conflicting, and weight reacquisition is considered a constant threat to the effectiveness of surgery. Irrespective of the type of bariatric procedure, many patients experience weight regain, which could range from a 46% to 63% increment over their lowest postoperative weight, during the first 2 years following surgery [7].

Freire et al. and Odom et al. have shown that the overall factors contributing to weight regain are multifaceted in their complexity. These factors have mainly been attributed to the dietary habits and lifestyle behaviors with the re-emergence of presurgery eating disturbances, excessive intake of calories, snacks, and sweets, and the psychological status of patients [8, 9]. Although they are known to play a major role in weight regain and maintenance, their exact contribution to weight regain remains to be further elucidated in patients who underwent bariatric surgery. In the present study, a comprehensive dietary and behavioral lifestyle assessment was carried out to explore this association and identify potential predictors of weight regain in patients following bariatric surgery.

## 2. Materials and Methods

### 2.1. Ethical Considerations and Informed Consent

All procedures and protocols were reviewed and approved by the Institutional Review Board at the College of Medicine, King Saud University. Written informed consent was obtained from all participants. This study was conducted at the Obesity Research Center, College of Medicine and King Khalid University Hospital, King Saud University, Riyadh, Saudi Arabia.

### 2.2. Study Design and Subjects

Fifty consecutive patients (56% female) who underwent bariatric surgery and completed an 18-month follow-up period postsurgery were recruited from the Obesity Clinic at the Obesity Research Center. Preoperative and postoperative clinical data and anthropometric measures were collected and recorded from patients during their follow-up visits at the clinic. Weight (in kilograms) was measured in light clothing and without shoes to the nearest 0.1 kg. Height was measured using a stadiometer, and body mass index (BMI) was calculated. Weight regain was calculated as the percentage increase of weight from the lowest postoperative weight to the present weight. Patients were stratified into weight maintainers (those who had < 15% weight regain, *n* = 29) and weight regainers (those who had ≥ 15% weight regain, *n* = 21). A dietary and behavioral lifestyle questionnaire was developed for the study purpose, from other validated questionnaires [9–12], into a single questionnaire targeting these behaviors which were applicable in our postbariatric patient population (Supplementary Table 1).

Questionnaire-based interviews were conducted by qualified clinical dietitians to assess the association between dietary habits, behavioral lifestyle practices, and weight regain. The assessment was carried out based on the patient's responses to each of the questions, which were scored within the two sections. Briefly, the scores were calculated for both sections, and the higher scores were considered as indicators of healthier dietary or lifestyle behaviors. A detailed subanalysis of dietary habits was also carried out to determine the patients' meal patterns and timing and their daily and weekly intake of frequently consumed foods and food groups.

### 2.3. Statistical Analysis

Statistical analysis was carried out using SPSS 22.0 (SPSS Inc., Chicago, IL, USA). Data are reported as the mean ± standard deviation (SD) for continuous variables, including age, height, weight, and BMI. Before the statistical analysis, a logarithmic transformation of the nonnormally distributed parameters was performed to approximate a normal distribution. Differences between the two groups (the weight maintainers and weight regainers) were assessed by an independent samples *t*-test. The association between the categorical variables was analyzed using the chi-square test. A *P* value of <0.05 was considered to be statistically significant. A subanalysis was carried out to evaluate if the gender of the patients influenced the weight regain in the overall group and also within the maintainers and regainers groups.

## 3. Results

### 3.1. Subjects and Anthropometric Characteristics

Both the groups included in the study were matched for age and BMI at the baseline (BMI, 48.9 ± 9.8 kg/m^2^ and 47.3 ± 8.9 kg/m^2^ for the maintainers and regainers, respectively, *P*=0.56). Both groups lost a significant amount of weight postoperatively, but weight regain was higher in the regainers (*P* < 0.001) (Table 1).

The maintainers group followed healthier dietary habits (44% vs. 10%, *P* ≤ 0.001) and healthier behavioral lifestyle practices (30% vs. 4%, *P* ≤ 0.002) than the regainers. The specific healthy dietary habits that were practiced by the maintainers group included (1) higher consumption of water, fruits, and vegetables, (2) limited intake of carbohydrates and fats, (3) regular consumption of breakfast, and (4) the consumption of more than five small frequent meals daily. The detailed healthy behavioral lifestyle practices that were practiced by the maintainers group included (1) practicing mindful grocery shopping behaviors, such as reading the nutritional label of products before buying them, (2) slowed and monitored pace of eating, (3) self-assessment behaviors, which included attending regular physician and nutritional follow-up visits and taking regular body weight measurements, (4) avoidance of negative eating behaviors, specifically late-night snacking, and (5) daily exercising for 20–30 minutes (Table 2). No statistically significant gender differences were seen in the overall group in relation to weight regain, although the male participants (*n* = 22) were seen more likely to regain more weight postoperatively than the female (*n* = 28) participants (15.3 ± 10.9 kg in male vs. 7.4 ± 6.9 kg in female, *P*=0.06). A similar trend was also noted in the weight regain pattern between both the maintainers (5.6 ± 3.4 kg in male vs. 4 ± 3.6 kg in female, *P*=0.26) and the regainers group (23.4 ± 7.7 kg in male vs. 16.9 ± 3.5 kg in female, *P*=0.058), with the males gaining more weight than their female counterparts but without statistical significance.

## 4. Discussion

Bariatric surgery is currently considered the most effective treatment for morbid and severe obesity and is being performed with an increasing frequency. In addition to the physiological changes, it also represents a forced behavioral modification that leads to significant changes in body weight. However, a constant threat to the success of bariatric surgery is weight regain, which indicates that close follow-up of patients postsurgery is important to retain the beneficial effects of surgery.

### 4.1. Overall Dietary Habits and Lifestyle Practices

Despite the initial successful weight loss seen in patients following surgery, the patients were found to regain approximately 11.3 ± 8.8% of their weight during the first 18 months of follow-up postsurgery, which was in line with previous reports [8, 13]. Among the fifty patients studied, 54% were found to follow healthy dietary habits, and 34% followed healthy behavioral lifestyle practices. This finding might indicate the predominance of unhealthy lifestyle behaviors among postbariatric surgery patients collectively compared to unhealthy dietary habits, stressing the fact that the two factors need to be considered as separate and independent causes for weight regain [8, 14].

### 4.2. Association of Dietary Habits and Weight Regain

Upon stratifying the patients into weight regainers and weight maintainers, we found a significant association between adherence to following healthy dietary habits and the maintenance of weight loss. The healthy dietary behaviors that stood out in the maintainers were (1) eating a regular breakfast, (2) having increased intake of water, and (3) eating foods with high dietary fiber content, which included vegetables and fruits. On the other hand, the eating choices of the regainers showed an increase in the consumption of carbohydrates and simple sugar-rich foods along with a limited intake of fiber and water (Table 2). Another dietary behavior that stood out in both groups was related to food selection, wherein postbariatric surgery patients showed a reduced preference towards eating high-fat containing foods and fast-food items. This finding might be explained by the patients' limited capacity to digest these foods postbariatric surgery [15]. It also further highlights the differences between postbariatric surgery patients who experience weight regain and obese patients in general, who on the contrary, tend to consume a high amount of fat-rich foods and fast foods. We also found that the regainers had a decreased fiber intake characterized by the complete elimination of fruits and vegetables from the daily diet along with low intake of water (Table 2). These findings illustrate that weight regain is more due to poor selection of healthy foods rather than excessive consumption, and thus, it is mainly attributed to food quality rather than quantity [16].

Patients who regained a significant amount of weight following surgery also showed differences in their meal pattern behaviors. The regainers consumed a limited number of meals, i.e., none of them ate more than 5 small frequent meals, including snacks, and usually skipped their breakfast. This finding might be explained by their false perceptions that reducing the number of meals helps lose more weight and maintain weight loss.

### 4.3. Effects of Behavioral Lifestyle Practices on Weight Regain

We found a significant association between healthy behavioral lifestyle practices and weight-loss maintenance. These practices were characterized by attending dietary counseling postsurgery with regular nutritional follow-ups, mindful grocery shopping behaviors, and self-assessment behaviors (Table 2). On the other hand, negative behavioral lifestyle practices included late-night snacking, emotional binge eating and distracted eating behaviors, and a lack of nutritional knowledge.

Nutritional follow-up visits are considered to be an essential component of medical management for patients following bariatric surgery. We found that patients who were compliant with their nutritional follow-up appointments were more likely to maintain their weight loss (*P* < 0.001) (Table 2). This finding is similar to what has been reported previously, where compliance with appointments was associated with a greater loss of excess body weight [17, 19]. Neglecting these visits would impact the patients' compliance with dietary advice and healthcare provider recommendations and fragments the dietitian-patient relationship. Additionally, these visits might serve as the only trusted source of nutritional knowledge for the patient, especially when other less-trusted sources are now abundantly available, where the accuracy and applicability of these sources are quite variable and can harmfully impact one's dietary or behavioral lifestyle practices [18]. We found an interesting difference between the two groups with regard to their food choices, as demonstrated by their grocery shopping, which was significantly different between the maintainers and regainers (*P*=0.001). The reported behaviors regarding grocery shopping revealed that only 14% of the regainers considered selecting foods based on healthy choices and studied the nutritional labels of products before buying them. In contrast, patients in the control group were more likely to consider healthy choices while shopping and properly utilized and understood food labels. This finding further confirms the importance of having prior nutritional knowledge of body weight maintenance among the postbariatric surgery patients. It is also crucial not to presume that a patient is “noncompliant” with the recommended dietary behaviors as they in fact may not have received the needed and updated dietary education [18]. Patients' nutritional knowledge should be assessed with high accuracy and detail, equivalent to the routine nutritional and medical assessment.

Active eating behaviors, such as stopping of eating with the first sensation of satiety, taking pauses between bites to measure fullness, practicing sufficient chewing while eating, and spending more than 20–30 minutes on meals, were seen more in the weight maintainers than in the weight regainers (Table 2). These results propose an association between active eating behaviors and weight loss maintenance. Postbariatric surgery patients who kept track of their eating pace were more likely to maintain their weight. Our findings were consistent with McGrice et al., who showed the essentiality of eating slowly and allowing a period of 20–30 minutes for meal consumption among postbariatric surgery patients [19]. On the other hand, disinhibited eating or negative eating behaviors, such as emotional binge eating (overeating in response to emotional stimuli or stress), distracted passive eating (defined as eating in the presence of cues that can trigger overeating and distract one's attention from the amount of food eaten), and late-night snacking, have been suggested to develop as a coping mechanism against negative emotions [20]. We found that both distracted passive eating (52% vs. 20%, respectively, *P*=0.02) and late-night snacking behaviors (57% vs. 17%, respectively, *P* < 0.003) were significantly higher in the regainers than in the maintainers. The predominance of these negative eating behaviors in the regainers further strengthened the strong association of these negative eating behaviors with postoperative weight regain [21, 22].

A healthy lifestyle incorporates both physical activity and exercise. We found that the maintainers were more physically active than the regainers (58% vs. 19%, respectively, *P* < 0.005). Among those who identified themselves as physically active, we found that 31% of maintainers and only 5% of regainers reported performing exercise for a period of 30 minutes or more on a daily basis. These findings also indicated a strong association between performing physical activity and weight loss maintenance among postbariatric surgery patients, which has also been shown previously [23]. To the best of our knowledge, this work is the first study that addressed weight regain in Saudi patients postbariatric surgery and comprehensively analyzed their specific dietary and behavioral lifestyle practices. The next step would be to confirm these findings in a larger cohort and implement an intervention to correct these negative behaviors to increase the health benefits of bariatric surgery.

## 5. Conclusion

Weight regain observed at the 18-month follow-up in postbariatric surgery patients was found to be mainly due to poor compliance to healthy dietary and behavioral lifestyle practices. These deficits in nutritional and behavioral practices need to be addressed through regular follow-ups and nutritional counseling sessions. Implementing these interventions will further ensure patient compliance, greatly impacting their degree of weight loss and facilitating its maintenance.

## Figures and Tables

**Table 1:** Characteristics of the study sample.

Characteristic	Weight maintainers (*n*=29)	Weight regainers (*n*=21)	*P*	Entire group (*n*=50)
Female/male (*n*)	19/10	9/12		28/22
Mean age (SD), years	41 (9.9)	41 (7.5)	0.799	41.4 (8.9)
Preoperative mean BMI (SD), kg/m^2^	47.3 (8.9)	48.9 (9.8)	0.564	48 (9.2)
Preoperative mean weight (SD), kg	127.7 (27.8)	135.4 (32.8)	0.376	131 (30)
Lowest mean weight reached postsurgery (SD), kg	79.4 (17.7)	77.8 (16.6)	0.753	78.7 (17.1)
Lowest mean BMI reached postsurgery (SD), kg/m^2^	29.5 (6)	28.2 (5.1)	0.431	29 (5.7)
Mean weight at the end of follow-up (SD), kg	84.1 (18)	96.6 (19.4)	0.023^*∗*^	89.3 (19.4)
Mean BMI at the end of follow-up (SD), kg/m^2^	31.2 (6)	35.8 (6.2)	0.011^*∗*^	33.1 (6.5)
Mean weight regain (SD), kg	5.4 (4.1)	19.5 (6.7)	<0.001^*∗*^	11.3 (8.8)

^*∗*^Significance *P* < 0.05.

**Table 2:** A detailed analysis of the differences in the dietary and lifestyle behaviors practiced between the weight maintainers and regainers.

	Weight maintainers (*n*=29)	Weight regainers (*n*=21)	*P*
Healthy dietary habits	44%	10%	<0.001

*Meal pattern behaviors*			
Breakfast consumption	23 (80%)	4 (19%)	0.001
Having three structured meals daily (breakfast, lunch, and dinner)	23 (80%)	5 (24%)	<0.001
Consuming more than five meals daily, including snacks	14 (48%)	0	<0.001

*Daily consumption of foods and food groups*			
Carbohydrates (1–5 Ex.)	18 (62%)	8 (38%)	0.094
Fat (3–5 Ex.)	17 (59%)	4 (19%)	0.005
Vegetables (3–5 Ex.)	18 (62%)	3 (14%)	0.001
Fruits (3–5 Ex.)	17 (59%)	1 (0.5%)	<0.001
Water (6–11 Cups)	23 (79%)	8 (38%)	<0.001

*Weekly consumption of foods and food groups*			
Ready-to-eat foods (0-1 time)	23 (79%)	13 (62%)	0.304
Fast foods (0-1 time)	24 (83%)	14 (67%)	0.189

Healthy lifestyle practices	30%	4%	0.002

*Grocery shopping behaviors*			
Consideration of healthy foods while shopping	27 (93%)	3 (14%)	<0.001
Selection of whole-grain over refined-grain products	17 (59%)	6 (29%)	0.035
Selection of low-fat products over full-fat products	20 (69%)	7 (33%)	0.013
Selection of foods based on nutritional facts	17 (59%)	2 (10%)	0.002
Reading the nutritional label of food products	22 (76%)	3 (14%)	<0.001

*Pace of eating*			
Stoppage of eating when feeling full	25 (86%)	12 (57%)	0.021
Taking pauses between bites	21 (72%)	5 (24%)	0.001
Taking >20–30 minutes for a meal	21 (72%)	4 (19%)	<0.001
Practicing sufficient chewing while eating	26 (90%)	10 (48%)	0.001

*Self-assessment behaviors*			
Regular body weight measuring	25 (86%)	6 (29%)	<0.001
Monitoring the daily consumed and burned calories	25 (86%)	4 (19%)	<0.001
Regular nutritional follow-up visits	15 (52%)	0	<0.001

*Negative eating behaviors*			
Emotional binge eating	7 (24%)	7 (33%)	0.475
Distracted eating	6 (21%)	11 (52%)	0.020
Late-night snacking	5 (17%)	12 (57%)	0.003

*Physical activity*			
Physically active	17 (59%)	4 (19%)	0.005
Daily exercise for ≥30 minutes	9 (31%)	1 (5%)	0.022

## Data Availability

All data generated or analyzed in the current study are included in this article.

## Supplementary Materials

Supplementary Materials Dietary and behavioral lifestyle questionnaire for postbariatric surgery patients.

## References

[1] Collaboration N. C. D. R. F. (2016). Trends in adult body-mass index in 200 countries from 1975 to 2014: a pooled analysis of 1698 population-based measurement studies with 19.2 million participants. *The Lancet*.

[2] Al Quwaidhi A. J., Pearce M. S., Critchley J. A., Sobngwi E., O’Flaherty M. (2014). Trends and future projections of the prevalence of adult obesity in Saudi Arabia, 1992-2022. *Eastern Mediterranean Health Journal*.

[3] Memish Z. A., El Bcheraoui C., Tuffaha M. (2014). Obesity and associated factors—kingdom of Saudi Arabia, 2013. *Preventing Chronic Disease*.

[4] Buchwald H., Williams S. E. (2004). Bariatric surgery worldwide 2003. *Obesity Surgery*.

[5] Alfadda A., Al-Dhwayan M., Alharbi A. (2016). The Saudi clinical practice guideline for the management of overweight and obesity in adults. *Saudi Medical Journal*.

[6] Colquitt J. L., Pickett K., Loveman E., Frampton G. K. (2014). Surgery for weight loss in adults. *Cochrane Database of Systematic Reviews*.

[7] Magro D. O., Geloneze B., Delfini R., Pareja B. C., Callejas F., Pareja J. C. (2008). Long-term weight regain after gastric bypass: a 5-year prospective study. *Obesity Surgery*.

[8] Freire R. H., Borges M. C., Alvarez-Leite J. I., Correia M. I. T. D. (2012). Food quality, physical activity, and nutritional follow-up as determinant of weight regain after Roux-en-Y gastric bypass. *Nutrition*.

[9] Odom J., Zalesin K. C., Washington T. L. (2009). Behavioral predictors of weight regain after bariatric surgery. *Obesity Surgery*.

[10] Gosadi I., Alatar A., Otayf M. (2017). Development of a Saudi Food Frequency Questionnaire and testing its reliability and validity. *Saudi Medical Journal*.

[11] Steinemann N., Grize L., Ziesemer K., Kauf P., Probst-Hensch N., Brombach C. (2017). Relative validation of a food frequency questionnaire to estimate food intake in an adult population. *Food & Nutrition Research*.

[12] Weineland S., Alfonsson S., Dahl J., Ghaderi A. (2012). Development and validation of a new questionnaire measuring eating disordered behaviours post bariatric surgery. *Clinical Obesity*.

[13] Harbottle L. (2011). Audit of nutritional and dietary outcomes of bariatric surgery patients. *Obesity Reviews*.

[14] Hudson S. M., Dixon J. B., O’Brien P. E. (2002). Sweet eating is not a predictor of outcome after lap-band. *Obesity Surgery*.

[15] Jeffery R. W., Baxter J., McGuire M., Linde J. (2006). Are fast food restaurants an environmental risk factor for obesity?. *International Journal of Behavioral Nutrition and Physical Activity*.

[16] Cena H., De Giuseppe R., Biino G. (2016). Evaluation of eating habits and lifestyle in patients with obesity before and after bariatric surgery: a single Italian center experience. *SpringerPlus*.

[17] El Chaar M., McDeavitt K., Richardson S. (2011). Does patient compliance with preoperative bariatric office visits affect postoperative excess weight loss?. *Surgery for Obesity and Related Diseases*.

[18] Johnson Stoklossa C., Atwal S. (2013). Nutrition care for patients with weight regain after bariatric surgery. *Gastroenterology Research and Practice*.

[19] McGrice M., Don Paul K. (2015). Interventions to improve long-term weight loss in patients following bariatric surgery: challenges and solutions. *Diabetes, Metabolic Syndrome and Obesity: Targets and Therapy*.

[20] Nyklíček I., Vingerhoets A., Zeelenberg M. (2011). *Emotion Regulation and Well-Being*.

[21] Pekkarinen T., Koskela K., Huikuri K., Mustajoki P. (1994). Long-term results of gastroplasty for morbid obesity: binge-eating as a predictor of poor outcome. *Obesity Surgery*.

[22] Chapman C. D., Benedict C., Jane Brooks S., Schiöth H. B. (2012). Lifestyle determinants of the drive to eat: a meta-analysis. *American Journal of Clinical Nutrition*.

[23] Bond D. S., Phelan S., Wolfe L. G. (2009). Becoming physically active after bariatric surgery is associated with improved weight loss and health-related quality of life. *Obesity*.

