# Supplementary material for: Dietary and Lifestyle Factors Serve as Predictors of Successful Weight Loss Maintenance Postbariatric Surgery
Source: J Obes. 2019 Feb 12;2019:7295978. doi: 10.1155/2019/7295978 (PMC6390255; doi:10.1155/2019/7295978)
Supplement: Supplementary Materials — Dietary and behavioral lifestyle questionnaire for postbariatric surgery patients. [file 7295978.f1.docx]

**Supplementary Table 1: Questionnaire**

| **Section 1: Dietary Habits. Theme 1: Consumption of Certain Foods and Food Groups** | |
| --- | --- |
| **Q1: Approximately how many exchanges of vegetable do you usually consume daily?** | None. 1 – 2 Ex. 3 – 4 Ex. ≥ 5 Ex. |
| **Q2: Approximately how many exchanges of fruit do you consume daily?** | None. 1 – 2 Ex. 3 – 4 Ex. ≥ 5 Ex. |
| **Q3:** **Approximately how many exchanges of carbohydrates do you consume daily?** | 1 – 5 Ex. 6 – 11 Ex. > 11 Ex. |
| **Q4: Approximately how many exchanges of fat do you consume daily?** | 1 – 2 Ex. 3 – 5 Ex. > 5 Ex. |
| **Q5:** **Approximately how many exchanges of dairy products do you consume daily?** | 1 – 2 Ex. 3 – 4 Ex. > 4 Ex. |
| **Q6: Approximately how many exchanges of legumes do you consume daily?** | None. 1 – 2 Ex. 3 – 4 Ex. ≥ 5 Ex. |
| **Q7: Approximately how many cups of water do drink daily?** | 2 – 4 Cups. 4 – 6 Cups. 6 – 8 Cups. 8 – 12 Cups. |
| **Q8: Which beverage do you usually drink between meals?** | Water. Fruit juices. Soft drinks. Coffee/Tea. |
| **Q9: How many times per week do you usually consume red meat?** | None. 1 – 2 Times 3 – 4 Times > 5 Times. |
| **Q10: How many times per week do you usually consume chicken?** | None. 1 – 2 Times 3 – 4 Times > 5 Times. |
| **Q11: How many times per week do you usually consume eggs?** | None. 1 – 2 Times 3 – 4 Times > 5 Times. |
| **Q12: How many times per week do you usually consume seafood?** | None. 1 – 2 Times 3 – 4 Times > 5 Times. |
| **Q13: In the last week, how many times did you eat sweets, candies, or cake?** | 0 – 1 Times 2 – 5 Times 6 – 10 Times > 10 Times. |
| **Q14: In the last week, how many times did you eat potato chips?** | 0 – 1 Times 2 – 5 Times 6 – 10 Times > 10 Times. |
| **Q15: In the last week, how many times did you eat processed meats?** | 0 – 1 Times 2 – 5 Times 6 – 10 Times > 10 Times. |
| **Q16: In the last week, how many times did you consume ready-to-eat foods?** | 0 – 1 Times 2 – 5 Times 6 – 10 Times > 10 Times. |
| **Q17: In the last week, how many times did you eat from a fast food restaurant?** | 0 – 1 Times 2 – 5 Times 6 – 10 Times > 10 Times. |
| **Q18: Are you taking any dietary supplements?** | Yes. Not anymore. Never. |
| If (Yes), the dietary supplements you are taking are | Iron. Vitamin B12. Vitamin C. Calcium.  Vitamin D. Zinc. Folic acid. Multivitamin.  Others (specify): ……… |
| **Section 1: Dietary Habits. Theme 2: Meal Pattern Behaviors** | |
| **Q1: How many meals, including snacks, do you usually consume daily?** | 1 – 2 Meals. 3 – 4 Meals. 5 – 6 Meals. > 6 Meals. |
| **Q2: How often do you consume breakfast on a regular basis?** | Always. Often. Sometimes. Never. |
| **Q3: How often do you have three structured meals (breakfast, lunch, and dinner)?** | Always. Often. Sometimes. Never. |
| **Q4: How often do you have a dessert after a meal?** | Always. Often. Sometimes. Never. |
| **Q5: Do you describe your diet as** | - Different each day. - Different sometimes during the week. - Different on the weekends. - Routine with specific guidelines. |
| **Q6: Your diet is mainly based on** | - Protein-rich foods (e.g., seafood, steaks, eggs, and dairy products). - Fat-rich foods (e.g., French fries, doughnuts, cookies, pastries, and other processed snacks). - Carbohydrate-rich foods (e.g., bread, cereals, pasta, rice, and potatoes). - Varied balanced diet. |
| **Q8: Your snacks usually consist of** | - Fruits and fruit juices, salads. - Potato chips, microwave popcorn, fried foods. - Biscuits, crackers, rusks, bread. - Sweets, chocolates, cakes, ice-creams. |
| **Section 1: Dietary Habits. Theme 3: Pace of Eating Behaviors** | |
| **Q1: Do you stop eating with the first sensation of satiety?** | Yes. Sometimes. No. |
| **Q2: Do you take pauses between bites to assess your fullness?** | Yes. Sometimes. No. |
| **Q3: Do you practice slow-eating behaviors (e.g., putting the spoon on the table after each bite)?** | Yes. Sometimes. No. |
| **Q4: Do you usually spend > 20 – 30 minutes while eating a meal?** | Yes. Sometimes. No. |
| **Q5: Do you practice sufficient chewing while eating?** | Yes. Sometimes. No. |
| **Q6: Do use small eating-utensils in meals (forks, spoons and plates)?** | Yes. Sometimes. No. |
| **Section 2: Lifestyle Practices. Theme 1: Grocery Shopping Behaviors** | |
| **Q1: Do consider healthy choices while grocery shopping?** | Yes. Sometimes. No. |
| **Q2: Do you usually choose whole-grain over refined-grain products?** | Yes. Sometimes. No. |
| **Q3: Do you usually choose low-fat/fat-free over full-fat products?** | Yes. Sometimes. No. |
| **Q4: Do usually read the nutritional label of products before buying them?** | Yes. Sometimes. No. |
| **Q5: Your food choices while shopping are based on** | - Personal preferences. - Nutritional facts. - Body tolerance. |
| **Section 2: Behavioral Lifestyle Practices. Theme 2: Self-assessment Behaviors** | |
| **Q1: Do you practice the same eating behaviors as you did before the surgery?** | Yes. Sometimes. No. |
| **Q2: Did surgery encourage you to change your previous eating habits?** | Yes. To some extent. No. |
| **Q3: After surgery, do you consider yourself more aware of your eating behaviors?** | Yes. To some extent. No. |
| **Q4: After surgery, do you consider yourself more aware of the fat and sugar content of foods?** | Yes. To some extent. No. |
| **Q5: After the surgery, do you consider yourself more capable of identifying nutrient-dense foods (i.e., foods that provide higher nutritional value than calories)?** | Yes. To some extent. No. |
| **Q6: Do you weigh yourself on a regular basis?** | Yes. No. |
| If (Yes), how often do you usually weigh yourself? | Daily. Weekly. Monthly. |
| **Q7: Do you monitor your consumed and burned calories?** | Yes. Sometimes. No. |
| **Q8: After surgery, did you encounter one or more of these behavioral changes?** | - Having more control over portion sizes. - Planning the amount of food to be eaten before actually eating it. - Being able to identify and avoid overeating cues and triggers. - Having more control over food urges. |
| **Q9: Do you have a dietitian?** | Yes. Not anymore. No. |
| If (Yes), do you consider yourself adherent to your nutritional follow-up visits? | Yes. Not anymore. No. |
| **Section 2: Behavioral Lifestyle Practices. Theme 3: Physical Activity** | |
| **Q1: Do you practice any physical activity on a regular basis?** | Yes. Sometimes. No. |
| If (Yes), how often do you practice it? | - Daily. - Five times a week. - 2 – 3 times a week. - Once a week. |
| If (Yes), for how long do you usually do it? | - < 10 minutes. - 10 - 20 minutes. - 20 - 30 minutes. - > 30 minutes |
| **Q2: Do you try to incorporate physical activity into your daily routine (i.e., using stairs instead of elevators)?** | Yes. Sometimes. No. |
| **Section 2: Lifestyle Practices. Theme 4: Negative Eating Behaviors** | |
| **Q1: Do you usually experience excessive eating episodes when feeling stressed or upset?** | Yes. Sometimes. No. |
| **Q2: Do you tend to practice late-night snacking to escape boredom, insomnia, or worried thoughts?** | Yes. Sometimes. No. |
| **Q3: Do usually eat in the presence of cues that can trigger overeating or distract your attention from the amount of food eaten (e.g., TV watching)?** | Yes. Sometimes. No. |
